# Study of corneal epithelial progenitor origin and the Yap1 requirement using keratin 12 lineage tracing transgenic mice

**DOI:** 10.1038/srep35202

**Published:** 2016-10-13

**Authors:** Ramesh Babu Kasetti, Subhash Gaddipati, Shifu Tian, Lei Xue, Winston W.-Y. Kao, Qingxian Lu, Qiutang Li

**Affiliations:** 1 Departments of Ophthalmology and Visual Sciences, University of Louisville School of Medicine, Louisville, KY 40202, USA; 2 Department of Interventional Radiology, Shanghai 10th People’s Hospital, Shanghai Key Laboratory of Signaling and Disease Research, School of Life Science and Technology, Tongji University, Shanghai 200092, China; 3 Department of Ophthalmology, University of Cincinnati, OH 45267, USA; 4 James Graham Brown Cancer Center, University of Louisville School of Medicine, Louisville, KY 40202, USA

## Abstract

Key issues in corneal epithelium biology are the mechanism for corneal epithelium stem cells to maintain the corneal epithelial homeostasis and wound healing responses, and what are the regulatory molecular pathways involved. There are apparent discrepancies about the locations of the progenitor populations responsible for corneal epithelial self-renewal. We have developed a genetic mouse model to trace the corneal epithelial progenitor lineages during adult corneal epithelial homeostasis and wound healing response. Our data revealed that the early corneal epithelial progenitor cells expressing keratin-12 originated from limbus, and gave rise to the transit amplifying cells that migrated centripetally to differentiate into corneal epithelial cells. Our results support a model that both corneal epithelial homeostasis and wound healing are mainly maintained by the activated limbal stem cells originating form limbus, but not from the corneal basal epithelial layer. In the present study, we further demonstrated the nuclear expression of transcriptional coactivator YAP1 in the limbal and corneal basal epithelial cells and its essential role for maintaining the high proliferative potential of those corneal epithelial progenitor cells *in vivo.*

Corneal epithelium serves as the surface barrier for the eye, which is constantly renewed with complete turnover for every 6–7 days^1^,^2^. In adults, corneal epithelial stem cells normally reside in the limbus at the cornea-scleral junction. They usually lack specific corneal differentiation markers, such as keratin-3 (Krt3) and keratin-12 (Krt12), and proliferate slowly^3^,^4^,^5^. Some of these limbal stem cells can divide asymmetrically to produce two different types of cells with one as a new generation of stem cell to maintain a constant stem cell population, and the other as a differentiating transit amplifying cell (TAC) that proliferates and migrates centripetally to populate the basal layer of the corneal epithelium^6^,^7^. These basal cells are able to divide continuously, or commit to cell cycle arrest as they migrate superficially and form suprabasal and superficial epithelial cells. During this process, the differentiating epithelial cells express stage-specific markers, e.g, Krt14 are restricted to the undifferentiated limbal and corneal basal cells; and the corneal type differentiation-specific Krt12 is expressed in the suprabasal cells and a portion of corneal basal cells^3^,^8^,^9^,^10^. It is suggested that the Krt12 expression in basal epithelial cells signifies the commitment of terminal corneal epithelium differentiation^10^. The terminally differentiated epithelial cells in most superficial layer form tight junctions as identified by expression of ZO-1 that is attributed to the barrier function of the corneal epithelium^11^,^12^. In the corneal epithelium, there appears to be a hierarchy of TACs with division capacity gradually reduced from periphery to central cornea^13^. It is most likely a hierarchy of TACs from early to late stages of the TACs formed sequentially.

However, recent studies using corneal transplant have challenged this model regarding the location of corneal stem cell and the pattern of TAC migration^14^, in which the corneal stem cells were suggested to distribute throughout the basal epithelial layer, and thus TACs were not necessary to migrate from limbus centripetally to replace the corneal epithelial cells during normal homeostasis^14^. Several genetic models have been used to study the limbal stem cell origin and fate, but these models have disadvantage in which many cell types can be initially marked during the development, and thus the location and behavior of one individual stem/progenitor cell are difficult to follow^15^,^16^. Therefore, the location of corneal stem/progenitors and the potential migration of TAC derived from these stem cells remains controversial^17^.

Corneal epithelial homeostasis is tightly controlled and regulated by interconnected pathways to keep the balanced cellular processes of proliferation, migration, and differentiation. However, mechanism in corneal epithelium progenitor cell maintenance and activation remains poorly characterized. Hippo signaling pathway has been demonstrated to maintain and support stem cell properties in various tissues, such as the liver, intestine, skin and nervous system^18^. This pathway is an evolutionarily highly conserved kinase cascade that controls cellular proliferation, differentiation and survival by integrating stimuli critical for tissue context-dependent development, including cellular density, mechanical stresses from tissue tension and stiffness, as well as metabolic cues^19^,^20^,^21^. Closely related Yap1 and TAZ proteins are the major downstream effectors of Hippo signaling pathway, and regulate target gene expression as transcriptional co-activators lacking intrinsic DNA binding activity. In general, YAP1 activity promotes proliferation of stem and progenitor cells by binding to other transcription factors, such as TEADs^18^,^22^. In addition, YAP and TAZ has been shown to increase cell motility and enhance neural crest cell migration^23^,^24^,^25^,^26^. While YAP has been implicated in maintenance of progenitor cell identity and proliferation, the precise role it plays in regulation of the corneal epithelial progenitor cell has not been characterized.

To overcome the early labeling of corneal stem cells during development and to selectively label the early stage of differentiating TACs, we have generated triple transgenic mice carrying three transgenes of *Krt12*^*rtTA*^*, TetO-Cre*, and *R26-floxSTOPflox-GFP* simultaneously*. Krt12* promoter activity determines the cell types to be labeled by Cre-mediated GFP expression after doxycycline (Dox) induction in this transgenic line. Mouse Krt12 appears at the corneal peridermal epithelium commenced at E13.5 during embryonic development, and is present in all cell layers of the adult corneal epithelium and suprabasal layers, but not in the basal layer of the limbal epithelium^10^,^27^. Interestingly, many basal epithelial cells, if not all, co-express both Krt 12 and Krt14 in adult cornea, in contrast to negative expression of Krt12 in many Krt14 positive basal cells in the young mice. Krt12 is generally regarded as a specific corneal epithelial differentiation marker, but it has also been observed to be induced in the stem/progenitor cells during their differentiation into the TACs^28^. In the present study, we showed that the limbal Krt12^+^- progenitor cells labeled by Dox-induced GFP survived up to 4 months; and when activated, could give rise to GFP-positive TACs that migrated centripetally to differentiate into corneal epithelial cells, and produced actinomorphic GFP tracking strips. The progenitor cells were located within the limbal niches during the normal homeostasis and small wound, but the large wound repair triggered the elongation of green strips accompanied with slight centripetal movement. Additionally, using this model we have also demonstrated that YAP1 is necessary for limbal progenitor cell proliferation and maintenance.

## Results

### Generation of *Krt12* ^*rtTA*^ */TetO-Cre/ROSA26-flox-STOP-flox-GFP* mice and Dox induction of the GFP expression

**Figure 1:**
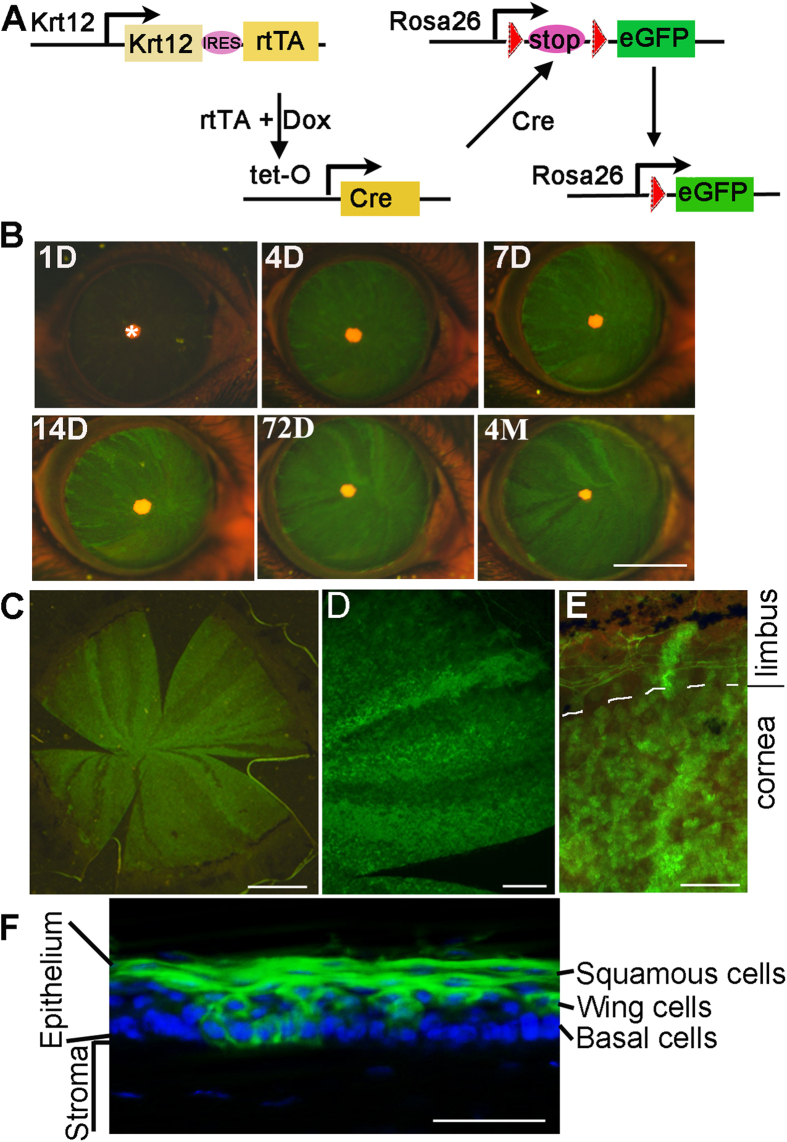
Generation of *Krt12*^*R/R*^*/TC/Rosa26*^*F/GFP*^ mice. (**A**) Schematic diagram of Krt12 promoter driven and Cre-mediated GFP expression. The Krt12 promoter drives the expression of rtTA. When bounded to Dox, rtTA/Dox turns on TetO-Cre expression in the Krt12^+^ cells. Cre recombinase then catalyzes the deletion of the loxP flanked STOP sequence to allow the expression of GFP in the Krt12^+^ lineages. Meanwhile, Cre activity also deletes *Yap1* exon 3 and abolishes the YAP1 protein expression. The *Krt12*^*R/R*^*/TC/Rosa26*^*F/GFP*^ mice at 1 month old were intraperitoneally injected with Dox at 80 μg/g body weight in 0.9% aqueous NaCl (10 mg/ml stock) followed by Dox-chow (1 g Dox/kg chow)^30^. (**B**) GFP expression at cornea is documented at indicated times after Dox treatment. *indicates the pupil under a fluorescence dissecting microscope. (**C**–**E**) Low (**C**) and high (**D**,**E**) magnification of GFP expression at whole mount corneal surface. (**F**) Corneal epithelium section shows GFP expression induced by Krt12/Cre. A mosaic pattern of GFP expression at cornea basal layer of *Krt12*^*R/R*^*/TC/Rosa26*^*F/GFP*^ mouse was observed at 7-days of Dox induction. Scale bars = 2 mm in (**B**), 1 mm in (**C**), 500 μm in (**D**), 100 μm in (**E**), 50 μm in (**F**). The images shown were representatives from 5 eyes examined.

In order to trace the corneal progenitor cell lineage and migrating track, we generated *Krt12*^*rtTA*^*(Krt12*^*R*^*)/TetO-Cre(TC)/ROSA26-flox-STOP-flox-GFP(Rosa26*^*F/GFP*^) (*Krt12*^*R*^*/TC/Rosa26*^*F/GFP*^) triple transgenic mice by breeding the *Krt12*^*R*^ mice with TetO-Cre and *Rosa26*^*F/GFP*^ mice (Fig. 1A). Krt12 is expressed predominantly by all differentiated suprabasal cells and some basal cells^10^,^28^. The *Krt12*^*rtTA*^ mice were created by knocking-in an IRES-reverse tetracycline transactivator (rtTA) cassette into the *Krt12* allele, under controlled by the endogenous *Krt12* promoter^10^,^29^,^30^. When bounded to Dox, the rtTA binds to TetO operator, and induces the expression of the Cre recombinase in the Krt12-positive cells^31^, which subsequently catalyzes the deletion of the loxP-flanked STOP sequence to allow expression of GFP^32^. This GFP expression permanently marks the progeny of Krt12-positive progenitor cells due to the ubiquitous CMV promoter that drives the GFP expression in the *Rosa26* locus^33^, until the progeny assume the terminal differentiation and slough off from epithelium.

With this *Krt12*^*R/R*^*/TC/Rosa26*^*F/GFP*^ triple transgenic mouse line, we first investigated the temporal and spatial expression patterns of the GFP marker. Since the *Krt12* promoter is predominantly activated in the differentiating suprabasal cells and some basal cells, we observed a large number of GFP-labeled cells throughout the corneal surface; and the number of GFP^+^ cells was gradually increased until day 4 of Dox induction (Fig. 1B–E). In contrast, in the absence of Dox or Krt12-driven rtTA, there was no GFP expressed (data not shown), suggesting that the GFP expression depended on Krt12 promoter-driven rtTA expression and Dox induction. Florescent microscopy showed that the GFP was expressed in the squamous cells and a subset of basal and wing cells, consistent with the predicted pattern of Krt12 promoter activity (Fig. 1F).

### The long term existence of the radial GFP^+^ stripes after Dox withdrawal suggests the presence of epithelial progenitor cells from KRT12 expressing lineage

**Figure 2:**
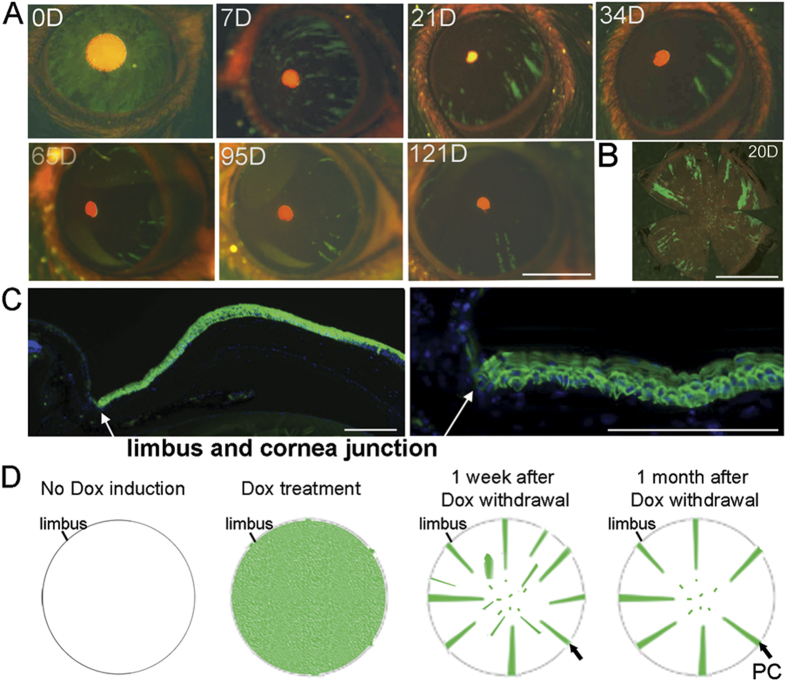
Long term presence of the radial GFP rays in *Krt12*^*R/R*^*/TC/Rosa26*^*F/GFP*^ mice after Dox withdrawal. GFP expression in *Krt12*^*R*^*/TC/Rosa26*^*F/GFP*^ triple mice at five weeks of age was induced by Dox for 4 days, then followed by regular chow without Dox. (**A**) Live images taken from a *Krt12*^*R*^*/TC/Rosa26*^*F/GFP*^ triple mouse at the indicated days after Dox withdrawal. (**B**) Flat mount image of cornea from a mouse at 20 days after Dox withdrawal. (**C**) Cryostatic cornea section showing a continuous GFP ray from a *Krt12*^*R*^*/TC/Rosa26*^*F/GFP*^ triple transgenic mouse. Diamidinophenylindole (DAPI; blue) was used for nuclear counterstaining. (**D**) Diagram showing dynamic change of Krt12/GFP expression and long-term presence of Krt12/GFP strips forming from Krt12/GFP marked limbal progenitor cells. Black arrows indicate the limbal epithelial progenitors. Scale bars = 2 mm in (**A**,**B**), 200 μm in (**C**). The images shown were representatives from 5 eyes examined.

To trace the differentiation pattern and lifespan of the GFP-labeled corneal epithelial progenitor/stem cells, we next withdrew Dox to turn off Cre expression and thereby halt the loxP mediated recombination in progenitor cells that commit terminal differentiation to assume corneal epithelial cell fate. As anticipated, the majority of GFP^+^ cells disappeared after 6–7 days, consistent with the known lifespan of the differentiated corneal epithelial cells^1^,^2^ (Fig. 2A). Surprisingly, a small subset of GFP^+^ cells persisted in the cornea for more than four months. It is worthy to note that these GFP^+^ cells were all organized in thin rays projecting from the peripheral limbal regions toward the central cornea (Fig. 2A,B). Histologically, these rays were composed of GFP marked basal cells and suprabasal cells (Fig. 2C). However, there were no limbal stem cells labeled as GFP positive. The GFP-positive ray stripe patterns most likely reflected the continuing proliferation, migration and differentiation tracks of the TACs that had been previously labeled by Cre-mediated expression of GFP before the withdraw of the Dox treatment (Fig. 2D). This is consistent with that the limbal stem cells do not express KRT12, but a subset of TACs directly adjacent to the limbus does^28^, suggesting that the GFP-labeled cells in the ray stripes were initially labeled in the TAC cells generated immediately from the limbal stem cells. Although the *Krt12*-promoter-dependent and GFP positive ray stripes existed up to 4 months, they almost completely diminished by 6 months after Dox withdrawal. This data suggests that the initial TAC cells generated from limbal stem cells can last for a life span of approximately 4 months, during which time they can maintain their population through asymmetric division, and meanwhile, generate the migrating and differentiating cells to participate in maintaining corneal epithelial homeostasis. However, due to their known propensity for limited level of symmetric division, the total population of GFP^+^-TACs is eventually completely exhausted in a period of approximately 4 months, and the new population of TAC needs to be continuously generated from limbal stem cells. Meanwhile, the GFP^+^-cells within the patches/stripes that disappeared within less than 4 months were most likely derived from later stages of TAC. No long-term surviving green patches in central cornea were detected.

### KRT12^+^ corneal epithelial progenitor cells contribute to wound healing response

Following injury, corneal epithelium is repopulated through proliferation and differentiation of the stem cells that are usually responsible for normal corneal epithelial homeostasis. Due to the latent nature of stem cells, the existence of corneal epithelial stem cells during regular corneal epithelia renewal is negligibly detectable. The wound-healing remodeling process offers an instant stem cell differentiation model allowing us to test whether a quiescent population of stem cells (or epithelial progenitor cells) exists in the corneal epithelium. To label the TACs with *Krt12*-promoter-Cre mediated GFP expression, we treated the *Krt12*^*R*^*/TC/Rosa26*^*F/GFP*^ mice at age of 5 weeks old with Dox for 4 days, and then suspended the Dox treatment for 3 weeks before performing the corneal epithelial injury. GFP stripes were monitored up to 1 month after injury.

**Figure 3:**
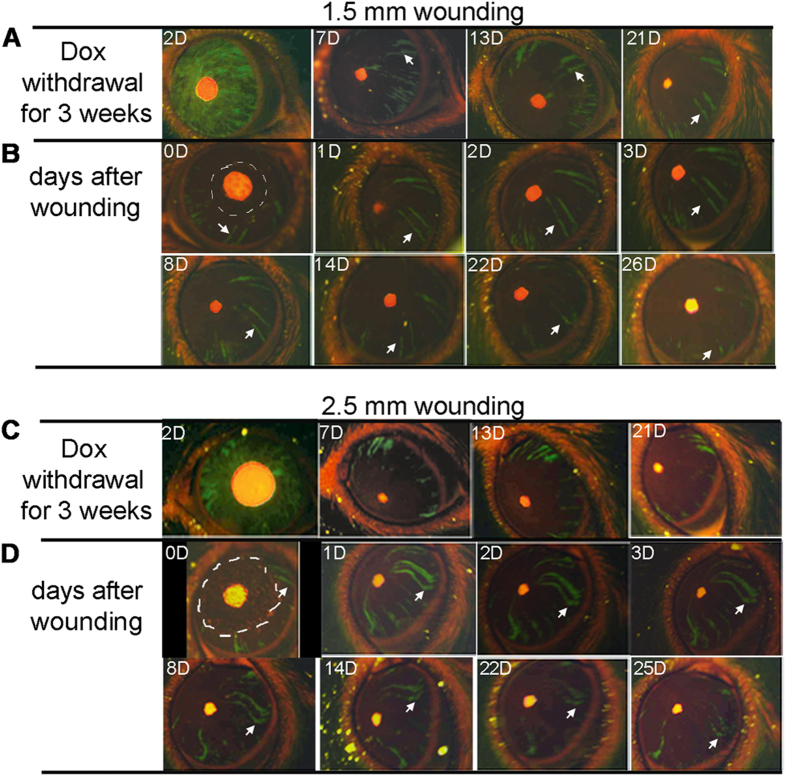
Response of limbal epithelial progenitor to corneal epithelial wounds. The *Krt12*^*R*^*/TC/Rosa26*^*F/GFP*^ triple mice at five weeks of age were injected with 80 μg/g body weight Dox in 0.9% aqueous NaCl (10 mg/ml stock) followed by Dox-chow (1 g Dox/kg chow) for 4 days, then fed with regular chow. The corneal epithelial injury was performed at 3 weeks after Dox withdrawal to ensure that only progenitor derived green strips were stabilized and observed. The green strip appearance and changes before and after wounding were recorded by photography. (**A**) Photographic images of the same eye at different days after Dox withdrawal. (**B**) Photographic images of the same eye at different days after 1.5 mm cornea epithelium debridement performed at 3 weeks after Dox withdrawal. The wound areas are outlined by white dashed lines. The white arrows point to a same green strip over the time. (**C**,**D**) Photographic images of another eye underwent a similar procedure as above, but wounded with 2.5 mm cornea epithelium debridement. The images shown are representatives from 5–6 eyes examined for each condition.

We performed corneal epithelial debridement with two different sizes. First, we removed part of cornea epithelium at a diameter of 1.5 mm within the central cornea, so that a dynamic progress that the GFP^+^ stripes grew and migrated to cover the wound area would be monitored. It is well established that corneal epithelial wound healing proceeds through three stages, characterized as an initial migratory stage to cover the wound with a two-cell layer epithelium, and followed by a proliferative stage to restore the stratified epithelium of 6–7 cell layers^34^. As shown in Fig. 3A,B, the GFP^+^-stripes extended to the injury area, and regrew back to original shape within the early healing period (1 day). After the wounded epithelium had been repaired, the stripes were still connected to the limbal regions, and this connection remained throughout 26 days.

It is suggested that the TAC cells or progenitors have a fixed division capacity. Large wounds might demand a large stem cell reserve, which may activate more stem cells and TAC progenitors at the early stage. To test the hypothesis, we performed the epithelium wounding with a larger area by a diameter of approximately 2.5 mm (Fig. 3C,D). Similar to the responses to the small wound, the GFP stripes were also elongated and extended to cover the injury area. However, the GFP-labelled population showed broken stripes and detachment from the limbus. Noticeably, the stripes that were detached from the limbus still kept its integrity for over a period of one month, but clearly shown with a shorter lifespan than the stripes in the uninjured eyes (Fig. 3D). This data suggested that wound repairing was carried out by the early TACs derived from the limbus, and this early TAC population had limited potential to proliferate, and could be exhausted by extensive wound healing process.

### Nuclear YAP1 was detected in corneal basal epithelial cells and limbus epithelial cells

**Figure 4:**
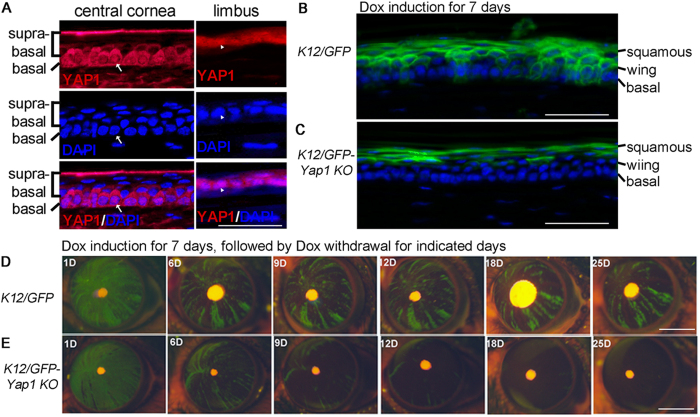
Requirement of YAP1 for maintenance of the cornea epithelial progenitors *in vivo*. (**A**) Nuclear YAP1 was detected in the limbal and corneal basal cells. DAPI was used for nuclear counterstaining. The arrows and arrowheads indicate the same position in the corneal and limbal epithelia, respectively. (**B**,**C**) Mosaic GFP expression at cornea basal layer of *Krt12*^*R/R*^*/TC/Rosa26*^*F/GFP*^ (*K12/GFP*) mouse at 7 week old (**B**), but absent in the age matched *Krt12*^*R/R*^*/TC/Rosa26*^*F/GFP*^*/Yap1*^*fl/fl*^ (*K12/GFP-Yap1KO*) mouse (**C**). The images were taken immediately after 7 days of Dox treatment. (**D**,**E**) Live images taken from a *Krt12*^*R/R*^*/TC/Rosa26*^*F/GFP*^ (*K12/GFP*) mouse (**D**) and *Krt12*^*R/R*^*/TC/Rosa26*^*F/GFP*^*/Yap1*^*fl/fl*^ (K12/GFP-*Yap1*KO) mouse (**E**) at the different time points after Dox withdrawal. The mice at five weeks of age were fed with Dox diet for 7 days, then followed by regular chow without Dox for indicated days. Scale bars = 35 μm in (**A**), 50 μm in (**B**,**C**), 2 mm in (**D**,**E**). The images shown are representatives from 5 eyes examined.

In attempt to identify genes that regulate stem cells identity and differentiation in corneal epithelium, we studied the YAP1 expression and intracellular distribution in the corneal epithelial cells. YAP1 is a downstream effector of the Hippo signaling pathway, and regulates stem cell maintenance and proliferation in a number of tissues^35^,^36^,^37^,^38^. To explore the potential role of YAP1 in corneal epithelium, we first examined the YAP1 distribution in mouse cornea, and found that YAP1 staining is mainly associated with corneal basal epithelial cells and limbus epithelial cells as well as some wing cells that immediately next to the basal cells (Fig. 4A). No YAP1 were detectable in terminally differentiated superficial squamous cells (Fig. 4A). YAP1 function is mediated by its subcellular localization controlled by its phosphorylation on several conserved residues^39^. The phosphorylation promotes YAP1 binding to 14-3-3 proteins and subsequent cytoplasmic sequestration. In corneal basal epithelial cells and limbus epithelial cells, both cytoplasmic and nuclear YAP1 were detected (Fig. 4A). Interestingly, only cytoplasmic but not nuclear localization YAP1 were observed in the wing cells (Fig. 4A). The distinct nuclear YAP1 localization suggests that YAP1 regulates critical cell processes during corneal epithelial progenitor differentiation.

### YAP1 is required for maintenance of the corneal epithelial progenitor cells

Nuclear localization of YAP1 in the corneal epithelial progenitor cells suggests a functional role it plays in these cells. To test the hypothesis, we deleted *Yap1* from the KRT12^+^ lineage by breeding the *Yap1*^*fl/fl*^ mice with the *Krt12*^*R/R*^*/TC/Rosa26*^*F/GFP*^ compound transgenic mice to obtain *Krt12*^*R/R*^*/TC/Rosa26*^*F/GFP*^*/Yap1*^*fl/fl*^ mice. Dox treatment for 7 days induced a GFP expression pattern in differentiated suprabasal corneal epithelial cells of the mutant mice as same manner as the WT mice, suggesting that YAP1 is not required for the survival and function of corneal squamous cells (Fig. 4B,C). The observation consists with the finding of YAP1 absence in corneal squamous cells (Fig. 4A). However, the number of GFP positive cells in the mutant basal layer was reduced (Fig. 4B,C), suggesting that YAP1 is required in the basal corneal epithelial cells.

**Figure 5:**
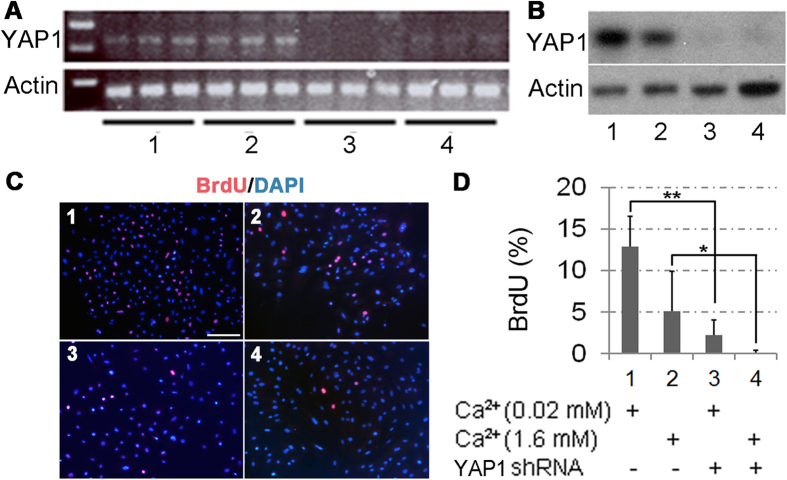
YAP1 regulation of the corneal epithelial cell proliferation *in vitro*. Primary mouse corneal epithelial cells were transduced with YAP1-specific (sample 3 and 4) or scramble control (sample 1 and 2) shRNA lentiviruses for 2 days; and then replaced with fresh medium containing 0.05 (sample 1 and 3) or 1.6 mM (sample 2 and 4) calcium and purimycin for additional 48 h. Data are representatives of three independent experiments. (**A**) RT-PCR showed that YAP1 was knocked down in primary corneal epithelial cells. (**B**) Loss of YAP1 expression in the shRNA knockdown cells was further confirmed by Western blot. (**C**) The representative images of BrdU staining (red) on the primary mouse cornea epithelial cells. Cells were pulse-labeled with BrdU for 90 min to label the proliferating cells. DAPI (blue) was used for nuclear counterstaining. (**D**) Quantitation of BrdU-positive cells. Data are expressed as mean ± SD. Statistical significance was analyzed by two-tailed Mann-Whitney U test: *P < 0.05, **P < 0.005. Scale bar = 150 μm in (**C**). The images shown were representatives from 3 independent experiments.

To further test the YAP1 function in regulation of TAC proliferation or survival, we tested the lifespan and the total numbers of the GFP-labeled basal cells after periods of Dox withdrawal. As shown in the Fig. 4D,E, deletion of *Yap1* demolished the GFP stripe formation with significantly reduction in the GFP^+^ cells in the *Yap1* mutant corneas as compared to the treated corneas in the *Yap1* WT mice, and no GFP strip survived over one month. These results suggest that YAP1 is critical for generation of the migrating cells from the corneal/limbal TAC to repopulate the central cornea. Since no apoptosis was evident within the *Yap1* KO corneas (results not shown), the lack of GFP^+^-stripes in the *Yap1* knockouts likely resulted from impairment in the initial division of the early TAC cells. To test this hypothesis, we knocked down the YAP1 expression in the mouse primary corneal epithelial cells with lentivirus expressing YAP1 shRNA. RT-PCR and Western blotting showed that YAP1 shRNA caused a dramatic reduction of YAP1 mRNA (Fig. 5A) and protein (Fig. 5B) by day 4 after transduction. Indeed, the YAP1 knockdown caused inhibition of the corneal epithelial cell proliferation (Fig. 5C,D). Blockage of the corneal epithelial proliferation by YAP1 knockdown was tested by BrdU incorporation, in which the BrdU-labeled proliferating cells were clearly detected in the WT epithelial cells, but significantly reduced in the YAP1 knockdown cells (Fig. 5D), further confirmed the notion that the YAP1 was required for proliferation of the corneal epithelial progenitor cells.

## Discussion

All self-renewing tissues including the cornea epithelium contain stem cells that have a unique capacity for both self-renewal and differentiation into all cell lineages derived from the same tissue origin. Adult stem cells are essential for tissue homeostasis and regeneration after injury. The loss of corneal transparency results in blindness of more than forty-five million individuals worldwide. For both conventional cornea transplant and emerging stem cell-based therapies for treating human cornea diseases, the scientific understanding stem cell behavior and regulation at both physiological condition (development and homeostasis) and pathological states (injury and degeneration) is a pressing need. However, lacking of specific permanent markers to identify and trace stem cells post the challenge for biological studies of the corneal epithelial stem cells.

Both clinical and scientific evidence suggest that the corneal epithelial stem cells are present at the limbus^17^. A well-accepted X, Y, Z hypothesis of corneal epithelial homeostasis has been developed, in which the movement of cells from the basal layers of the corneal epithelium (X) and the movement of cells from the periphery of the cornea (Y) replace those cells that are lost from the corneal surface through natural shedding (Z)^40^. However, recent study reported a controversy result that, in addition to the limbal epithelium, the corneal epithelial stem cells also resided within corneal epithelium^14^. To precisely label the differentiating corneal progenitor cells temporally and spatially, we have generated transgenic mice in which the *Krt12* promoter-driven rtTA transgene and Dox-dependent expression of Cre (tet-O-Cre) that turns on GFP expression in corneal epithelial cells derived from KRT12 positive basal corneal TAC and limbal progenitor cells, therefore, the survival duration, migration pattern, and lineage tracing of the GFP-labeled progenitor cells can be tracked. As expected, Dox treatment induced GFP expression in corneal epithelial cell in these compound transgenic mice. Dox withdrawal turned off the Cre expression and thereby halted the flox-mediated recombination in the early stages of corneal epithelial progenitor cells that had not yet activated the Krt12 promoter activity. As the differentiation continues, these newly differentiating cells remained GFP negative due to the lack of Dox-depended expression of Cre; and as a result, the majority GFP positive cells that had been previously labeled before the withdrawal of Dox disappeared within 6–7 days, consistent with the known lifespan of differentiated corneal epithelial cells^1^,^2^. But surprisingly, a subset of GFP positive cells persisted in the cornea for more than four months after Dox withdrawal, as shown in the present study. Interestingly, all these GFP-positive cells were organized in thin rays projecting from limbus into the central cornea. Histologically, these rays were derived from the GFP marked progenitor cells, TAC/progenitor cells (KRT14^+^ and KRT12^+^), and differentiating epithelial cells. We then concluded that this GFP pattern reflected a mosaic pattern of limbal stem/progenitor labeled with GFP, and the ray patterns represented centripetal migration of the TACs derived from a limbal stem/progenitor cell. The progenitor cells were located within the limbal niches during normal homeostasis and small wounding, but the large wound repair triggered the elongation of green strips accompanied with slight centripetal movement. Our data supports the notion that corneal epithelial homeostasis and wound healing are mainly maintained by the activated limbal stem cells but not by the progenitor cells existing in the corneal basal layer. Our work further corroborates recent observations on the important role of corneal epithelial stem cells and progenitors in corneal epithelial homeostasis and wound healing using Krt14 lineage tracing system^41^,^42^,^43^. With our cell lineage tracking system, we did not detect any progenitor cells within corneal epithelium, since no GFP green patches or stripes were observed in the central cornea. The discrepancy in the stem cell properties has been revealed between the genetic lineage-tracing *in vivo* and transplantation assay^44^. It is possible that the extrinsic factors that are induced by tissue grafting in the previous study might activate the corneal epithelial stem cells resided in corneal epithelium^14^.

We further reasoned that this model would allow us to evaluate the genes that regulated corneal epithelial stem/progenitor generation and function by examining the green ray generation and maintenance. Due to intrinsic complexity and difficulties in characterization of the regulatory networks in the corneal stem cells, little is known regarding the molecular networks controlling corneal stem/progenitor maintenance, proliferation, and differentiation. Only few factors, such as p63^45^,^46^ and Pax6^47^, Tcf4^48^, have been implicated as essential factors for maintenance of the long-term proliferative capacity of the epidermal stem cells *in vivo*. Our corneal stem/progenitor cells tracking system revealed a mosaic pattern of GFP labeling, each stripe ray represents the progenies from an individual stem/progenitor cell, which allows us to examine the interruption of the proliferation, migration, or differentiation for a differentiating progenitor cells and evaluate a gene function in such regulatory pathways. YAP1 is a well-studied transcriptional coactivator in the conserved Hippo kinase cascade and required for stem cell maintenance and proliferation in a number of epithelium tissues, where it acts as a coactivator with TEAD to regulate several stemness genes^19^,^39^,^49^,^50^,^51^,^52^. Loss of YAP1 in the embryonic neuroepithelium led to decreased progenitor cell survival^36^, and *Yap1* inactivation in the embryonic epidermis reduced progenitor cell proliferation^37^. In contrast, loss of YAP1 in the intestinal epithelium caused no obvious phenotype at normal physiological conditions, but resulted in hyperplasia and increased stem cell replication after injury^53^. Thus, YAP1 in epithelial tissues acts in a tissue-, cell-, and context dependent manner. To elucidate the function of YAP1 in cornea epithelium, we first examined its expression pattern and found that YAP1 was constantly present in both cytoplasm and nucleus in the central and limbal basal cells. Deletion of YAP1 from corneal progenitors abolished GFP-labeled stripe formation originated from the limbal progenitor cells. This is consistent with the *in vitro* study that, when lacking YAP1, the proliferation of the primary corneal epithelial cells is significantly reduced. Our present study using loss-of-function mutation clearly shows that YAP1 is a critical regulator for corneal epithelial stem cell and progenitor proliferation.

Understanding of the YAP1 function and its interaction with other components of Hippo pathway in regulation of corneal epithelial stem cell proliferation and differentiation may uncover novel therapeutic strategies for treating corneal wound injury; and availability of small molecules designed to target Hippo signaling^54^ may provide potential for development of topical medications for treating corneal diseases. Our mouse model developed in this investigation has been proven to be useful for identification of the genes that mediate corneal epithelial stem/progenitor function, and can be adopted for study of many other genes that function in corneal stem/progenitor cells.

## Methods

### Animals

*Krt12*^*rtTA*^ mice were created by knock-in of the reverse tetracycline-controlled transactivator (rtTA) including an internal ribosome entry site sequence into one *Krt12* allele^10^,^29^,^30^. *TetO-Cre* mice (stock # 006224) *and ROSA26-flox-STOP-flox-GFP* mice (stock #007676) mice were purchased from the Jackson Laboratory. *Yap1*^*fl/fl*^ mice were purchased from International Knockout Mouse Consortium. Experimental animals were housed under pathogen-free conditions and handled in accordance with guidelines approved by the Institutional Animal Care and Use Committee of the University of Louisville.

Dox induction was performed by intraperitoneal injection with 80 μg Dox/g body weight in 0.9% aqueous NaCl followed by Dox-chow (1 g Dox/kg chow)^30^.

### Cornea imaging

GFP expression in the cornea surface was monitored using a Zeiss Discovery 8 fluorescent stereomicroscope equipped with the Zeiss GFP wideband cube (Zeiss KSC 295-831D, excitation HQ470/440 nm). The digital images of frontal views of corneas were captured with an advance digital camera.

### Cornea epithelium debridement

Mice were anesthetized with an intraperitoneal injection of ketamine (50 mg/kg) and xylazine (5 mg/kg). After a few drops of 0.5% topical proparacaine were applied, the central epithelium, excluding the limbal area, was demarcated with a 1.5 mm- or 2.5 mm-diameter biopsy punch, and removed by gently scraping with a blade^55^. Such wound only removes the epithelium and leaves the intact basement membrane. At each time point or condition, five to six animals were analyzed.

### Histology and immunostaining

Eyes were fixed in 4% PFA at 4 °C for overnight, and then subjected to OCT or paraffin embedding and sectioning for histological studies. The paraffin-embedded sections (7 μm thick) were prepared for hematoxylin and eosin (H&E) staining. For most immunostaining, the tissue sections or cultured cell preparations were subjected to an antigen-retrieval procedure by heating the slides at 95 °C for 30 min in 10 mM Tris-ethylendiaminetetraacetic acid (EDTA) buffer (pH 9.0). The primary antibodies used in this study were chicken anti-GFP (1:200, cat #GFP-1020, Aves Lab, Inc., Tigard, OR), rat anti-BrdU (1:800, MAS 250c, Harlan-Sera Lab, Belton Loughborough, Leicestershire, England), and mouse anti-YAP1 (1:100, cat. # 56701, Abcam). The secondary antibodies, conjugated with either carbocyanine 3 (Cy3) or fluorescein isothiocyanate (FITC), were purchased from Jackson ImmunoResearch Laboratories, Inc. (West Grove, PA).

### Corneal epithelial cell culture

Eyes were collected from euthanized mice and treated for overnight at 4 °C for with dispase II (10 mg/ml in DMEM medium) to disrupt the basement membrane, then the epithelial sheets were peeled off and digested in 0.25% trypsin-EDTA at 37 °C for 5–10 min. The cells were washed in Dulbecco’s modified Eagle’s medium (DMEM) containing 10% fetal bovine serum (FBS) [HyClone] and resuspended in keratinocyte serum-free medium (KSFM; Invitrogen) containing 0.05 mM calcium and plated on collagen-coated tissue culture plates. To induce differentiation, the cultures were exposed to 1.6 mM calcium for 48 h, then were fixed in 4% paraformaldehyde (PFA) for immunostaining or collected for Western analysis or qPCR analysis.

### Lentiviral vector, viral production, and viral transduction

Expression vectors encoding the YAP1 shRNA and scrambled control shRNA were purchased from Open Biosystems, Inc. Lentivirus was produced by a four-plasmid (for 3^rd^-generation lentiviral vectors) transfection system as described by Tiscornia *et al.*^56^. Lentivirus was collected in keratinocyte serum-free medium and directly used to transduce primary corneal epithelial cells for overnight.

### Calculation of the number of BrdU-positive cells and statistical analysis

In the YAP1 knockdown experiments, the number and percentage of BrdU-positive cells for 1000 randomly selected YAP1 shRNA lentivirus-transduced cells and for 1000 control lentivirus-transduced cells were determined in three independent experiments. All data are summarized as the mean ± standard deviation (SD). Two-tailed Mann-Whitney U test was performed to determine the significance of the differences between means. The differences were considered statistically significant if the *p* values were less than 0.05.

### RNA isolation, quantitative (q) PCR, and Western blot analysis

The experimental methods were previously described^57^. The primers for mouse *Yap1* (ID 15928514a1) were designed based on the online PrimerBank database (Harvard Medical School, Boston, MA; http://pga.mgh.harvard.edu/primerbank). The mouse anti-YAP1 antibody (1:100, cat # sc-101199) was purchased from Santa Cruz Biotechnology, and mouse anti-β-actin antibody (1:1000, cat #A2228) was purchased from Sigma.

## Additional Information

**How to cite this article**: Kasetti, R. B. *et al.* Study of corneal epithelial progenitor origin and the Yap1 requirement using keratin 12 lineage tracing transgenic mice. *Sci. Rep.* **6**, 35202; doi: 10.1038/srep35202 (2016).
